# Cancer Epigenetic Biomarkers

**DOI:** 10.3390/cancers15225365

**Published:** 2023-11-10

**Authors:** Fabio Coppedè

**Affiliations:** 1 Department of Translational Research and of New Surgical and Medical Technologies, University of Pisa, 56126 Pisa, Italy; fabio.coppede@unipi.it; Tel.: +39-050-2218544; 2 Interdepartmental Research Center of Biology and Pathology of Aging, University of Pisa, 56126 Pisa, Italy

This series of nine articles (six original articles, three reviews) is presented by international experts in cancer epigenetics. Cancer is among the most threatening human diseases, with millions of diagnosed novel cases and deaths occurring worldwide every year. The global aging of the population and the increased exposure to environmental carcinogens, coupled with the adoption of lifestyle behaviors such as smoking and alcohol drinking, poor diets, and scarce physical activity, account for the pathogenesis of most cancers in both developed and less-developed countries. It has now been ascertained that genetic, epigenetic, and cytogenetic modifications occur within cancer cells and tissue, many of which are driven by environmental exposure and are responsible for the acquisition of the malignant phenotype. Particularly, it is now evident that hundreds of genes alter their expression in the multistep process of carcinogenesis due to epigenetic events, including promoter hypermethylation, histone tail modifications, chromatin remodeling, or mechanisms mediated by non-coding RNA molecules. Some of these epigenetic modifications are garnering enhanced interest in the clinical setting as potential diagnostic or prognostic biomarkers of disease, as well as response predictors to therapy. This Special Issue focuses on recent advances in cancer epigenetics, as well as on the discovery and utility of cancer epigenetic biomarkers.

Two articles in this Special Issue investigate DNA methylation in lung cancer. Larsen et al. [1] focus their research on understanding the molecular mechanisms that mediate treatment response in non-small-cell lung cancer. Indeed, a treatment option for patients with non-small-cell lung cancer relies on immunotherapy, which targets the interaction between programmed cell death protein 1 (PD-1) and programmed death-ligand 1 (PD-L1). The effectiveness of this treatment depends on the expression levels of PD-L1 in tumor cells. The authors investigate whether PD-L1 expression levels depend on *PD-L1* gene promoter methylation levels, observing only a weak inverse correlation between *PD-L1* methylation and expression levels in tumor biopsies [1]. Diakofotaki et al. [2] explore transcriptomic and methylomic datasets from both human lung adenocarcinoma cell lines and the Cancer Genome Atlas (TCGA), observing that global genome hypomethylation in lung adenocarcinoma is associated with the increased expression of specific gene clusters, including germline-specific genes, genes expressed in the gastrointestinal tract, and genes expressed in stratified epithelia. Furthermore, the expression levels of some genes in the latter group are found to be associated with the poor survival of lung cancer patients [2]. In their study, Tournier and coworkers [3] screen tumor biopsies from stage II colon cancer patients and reanalyze data from TCGA-derived cohorts of stage II colon cancer patients to identify methylomic signatures of clinical utility. Their analysis identifies four previously undescribed molecular sub-classes of stage II colon cancer and suggests that the methylation levels of *CDH17* and *LRP2* genes may be able to predict the risk of cancer recurrence [3]. Vay et al. [4] investigate the expression levels and prognostic significance of the inhibitor of apoptosis protein survivin in pancreatic ductal adenocarcinoma, observing that the cytoplasmic and nuclear overexpression of survivin is associated with advanced disease stages and poor prognosis.

In addition to the analysis of DNA methylation in cancer tissues, the search for circulating epigenetic biomarkers of clinical utility is very attractive as they can be obtained using minimally invasive procedures. Two articles in this Special Issue investigate the potential clinical utility of circulating DNA methylation biomarkers. The research article by Fu and coworkers [5] investigates the association between the methylation levels of imprinted genes in peripheral blood cells and the risk of breast cancer. To this end, the authors use methylomic data available from the TCGA and Gene Expression Omnibus (GEO) datasets to the identify CpG sites of imprinted regions associated with breast cancer and conduct an independent case–control study in over 1000 individuals to validate their findings. The study reveals that the decreased methylation of *KCNQ1* and *KCNQ1OT1* and the increased methylation of *PHLDA2* are associated with an increased risk of breast cancer. Furthermore, it is found that the methylation of the *KCNQ1OT1* region is not affected by leukocyte heterogeneity, suggesting that it may represent a good candidate for breast cancer risk assessment [5]. In their study, Bos and coworkers [6] define a panel of renal-cell carcinoma DNA methylation biomarkers from circulating cell-free DNA that appear to be associated with shorter progression-free survival. The study paves the way for larger prospective studies aiming to validate whether circulating cell-free DNA methylation can be employed to identify patients at high risk of rapid disease progression.

Three interesting review articles are included in this Special Issue. The first deals with the epigenetic biomarkers of drug resistance [7]. Platinum-based chemotherapy is the first-line treatment for a variety of cancers, but its effectiveness is often limited by the acquisition of tumor resistance. DNA methylation-based biomarkers are increasingly investigated as potential biomarkers for the selection of patients who may or may not benefit from chemotherapy. To this end, Tavares and coworkers perform a systematic review of articles describing DNA methylation biomarkers that could be predictive of resistance to platinum-based chemotherapy, highlighting current limitations to their clinical application and future directions [7]. Thymic epithelial tumors (TETs) are rare malignancies of the anterior mediastinum, and include thymomas, thymic carcinomas, and neuroendocrine tumors. Genetic and cytogenetic investigations in TETs have enabled the main molecular subtypes to be defined, but the epigenetic landscape of these tumors remains far from being completely elucidated. Changes in DNA methylation and non-coding RNAs have been investigated in thymomas and thymic carcinomas, with less attention paid to the post-translational modification of histone tails and to the epigenetic changes that occur in neuroendocrine tumors. An updated review of the proposed epigenetic biomarkers of TETs is included in this Special Issue [8]. Histone chaperones are required for histone storage, transport, post-translational modifications, nucleosome assembly, and histone turnover. The third review in this Special Issue describes the role of selected histone chaperones in the progression of digestive cancers and their prognostic significance [9].

Collectively, this collection of original research articles and updated reviews provides a broad overview of cancer epigenetic biomarkers, highlighting their potential to contribute to significant advances in cancer biology and treatment; this includes optimizing the definition of cancer molecular subtypes, the early identification of individuals at risk of cancer onset, recurrence or rapid progression, and enhancing the design of personalized therapeutic approaches.
